# (2*S*,3*S*)-3-(4-Chloro­phen­yl)-8-methyl­tropane-2-carboxylic acid

**DOI:** 10.1107/S1600536808025002

**Published:** 2008-08-09

**Authors:** Zheng-Ping Chen, Song-Pei Wang, Xiao-Min Li, Jie Tang, Jian-Guo Lin

**Affiliations:** a The Key Laboratory of Nuclear Medicine, Ministry of Health, Jiangsu Institute of Nuclear Medicine, Wuxi 214063, People’s Republic of China

## Abstract

In the title compound, C_15_H_18_ClNO_2_, the inter­nal torsion angles of the tropane ring are comparable to those of tropane rings in the crystal structures reported for cocaine and its derivatives. There is an intra­molecular hydrogen bond between the N atom in the tropane ring and the O atom of the carboxyl group. The crystal structure is further stabilized by many weak C—H⋯O inter­actions between the mol­ecules in the *ab* plane, forming a two-dimensional supra­molecular network.

## Related literature

For general background, see: Clarke *et al.* (1973 ▶); Carroll *et al.* (1991 ▶, 2005 ▶). For related structures, see: Meltzer *et al.* (1997 ▶, 2001 ▶); Zhu *et al.* (1999 ▶). For related literature, see: Meegalla *et al.* (1997 ▶). For a description of the Cambridge Structural Database, see: Allen (2002 ▶). 
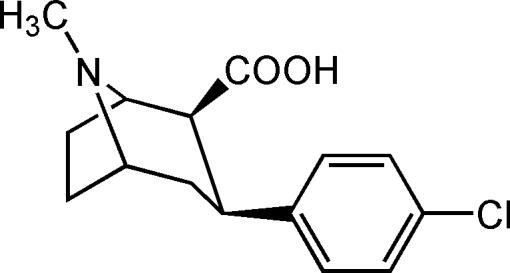


## Experimental

### 

#### Crystal data

C_15_H_18_ClNO_2_
*M* *_r_* = 279.75
Monoclinic, 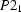

*a* = 8.219 (6) Å
*b* = 6.501 (4) Å
*c* = 12.731 (8) Å
β = 100.692 (10)°
*V* = 668.4 (8) Å^3^
*Z* = 2
Mo *K*α radiation
μ = 0.28 mm^−1^
*T* = 293 (2) K
0.20 × 0.10 × 0.10 mm

#### Data collection

Bruker SMART APEX CCD diffractometer
Absorption correction: multi-scan (*SADABS*; Bruker, 2001 ▶) *T*_min_ = 0.956, *T*_max_ = 0.976
3374 measured reflections
2760 independent reflections
2264 reflections with *I* > 2σ(*I*)
*R*_int_ = 0.042

#### Refinement

*R*[*F* ^2^ > 2σ(*F* ^2^)] = 0.052
*wR*(*F* ^2^) = 0.125
*S* = 0.99
2760 reflections
177 parameters
2 restraints
H atoms treated by a mixture of independent and constrained refinement
Δρ_max_ = 0.25 e Å^−3^
Δρ_min_ = −0.21 e Å^−3^
Absolute structure: Flack (1983 ▶), 1135 Friedel pairs
Flack parameter: −0.15 (9)

### 

Data collection: *SMART* (Bruker, 2001 ▶); cell refinement: *SAINT* (Bruker, 2001 ▶); data reduction: *SAINT*; program(s) used to solve structure: *SHELXTL* (Sheldrick, 2008 ▶); program(s) used to refine structure: *SHELXTL*; molecular graphics: *SHELXTL*; software used to prepare material for publication: *SHELXTL*.

## Supplementary Material

Crystal structure: contains datablocks global, I. DOI: 10.1107/S1600536808025002/fj2136sup1.cif

Structure factors: contains datablocks I. DOI: 10.1107/S1600536808025002/fj2136Isup2.hkl

Additional supplementary materials:  crystallographic information; 3D view; checkCIF report

## Figures and Tables

**Table 1:** Hydrogen-bond geometry (Å, °)

*D*—H⋯*A*	*D*—H	H⋯*A*	*D*⋯*A*	*D*—H⋯*A*
N10—H10*X*⋯O1	0.881 (17)	1.80 (2)	2.599 (3)	150 (3)
C9—H9⋯O1^i^	0.98	2.28	3.148 (4)	146
C16—H16*A*⋯O2^ii^	0.96	2.48	3.282 (4)	141
C14—H14*A*⋯O2^iii^	0.97	2.54	3.439 (4)	154

Symmetry codes: (i) 
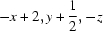
; (ii) 
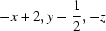
; (iii) 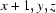.

**Fig. 1.**
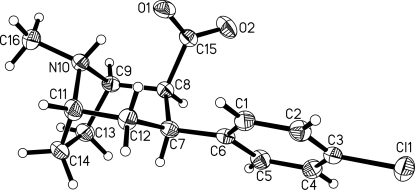
The molecular structure of (I), with atom labels and 50% probability displacement ellipsoids for non-H atoms.

**Fig. 2.**
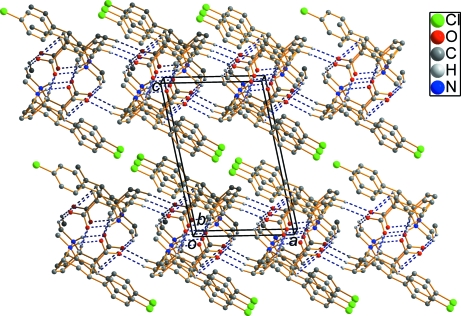
Perspective view of the supramolecular network along ab plane built from intermolecular weak C—H···O hydrogen bonding interactions (dashed lines). H atoms not involved in hydrogen bonding have been omitted.

## supplementary crystallographic information

### Comment

(2*S*,3S)-3-(4-halogen-phenyl)tropane-2-carboxylic acid methyl ester and analogues, the so-called "WIN compounds" reported by Clarke *et al.* (1973), been used extensively in medicine as monoamine uptake inhibitors and dopamine transporter (Carroll *et al.*, 1991, 2005). Among these, only several crystal structures have been reported (Meltzer *et al.*, 1997, 2001; Zhu *et al.*,1999) (Cambridge Structural Database, Version 5.29, update of November 2007; Allen, 2002). As a vital intermediate compound for the stepwise reactions of dopamine transporter-imaging agent, the crystal structure of the title compound, (I) (Fig. 1), has not been studied yet. The internal torsion angles of the tropane ring in (I) are comparable to those tropane rings in the crystal structures reported for cocaine and its derivatives. There is an intramolecular hydrogen bond between the N10 atom in the tropane ring and O1 atom of the carboxylate group (Table 1). The crystal structure is further stabilized by many weak C—H···O interactions between the intramolecules along *ab* plane to form two-dimensional supramolecular network.(Fig. 2 and Table 1).

### Experimental

Compound (I) was synthesized according to the method reported in the literature (Meegalla *et al.*,1997). A white powder was obtained (yield 41%) and was recrystallized from a mixed solvent composed of acetone, methanol and ether (1:1:1 *v*/*v*/v); white block-shaped crystals were obtained after several days (yield 36%). Analysis calculated for C_15_H_18_ClNO_2_: C 64.40, H 6.95, N 5.01%; found: C 64.17, H 6.98, N 4.90%.

### Refinement

H atoms bonded to N atom was located in a difference map and refined with distance restraints of N—H = 0.881 (17) Å, and with *U*_iso_(H) = 1.2*U*_eq_(N). Other H atoms were positioned geometrically and refined using a riding model (including free rotation about the ethanol C—C bond), with C—H = 0.93–0.98 Å and with *U*_iso_(H) = 1.2 (1.5 for methyl groups) times *U*_eq_(C).

### Crystal data

**Table:**

C_15_H_18_ClNO_2_	*F*_000_ = 296
*M**_r_* = 279.75	*D*_x_ = 1.390 Mg m^−^^3^
Monoclinic, *P*2_1_	Mo *K*α radiation λ = 0.71073 Å
Hall symbol: P 2yb	Cell parameters from 742 reflections
*a* = 8.219 (6) Å	θ = 3.3–26.8º
*b* = 6.501 (4) Å	µ = 0.28 mm^−^^1^
*c* = 12.731 (8) Å	*T* = 293 (2) K
β = 100.692 (10)º	Block, white
*V* = 668.4 (8) Å^3^	0.20 × 0.10 × 0.10 mm
*Z* = 2	

### Data collection

**Table:**

Bruker SMART APEX CCD diffractometer	2760 independent reflections
Radiation source: fine-focus sealed tube	2264 reflections with *I* > 2σ(*I*)
Monochromator: graphite	*R*_int_ = 0.042
*T* = 293(2) K	θ_max_ = 27.2º
φ and ω scans	θ_min_ = 1.6º
Absorption correction: multi-scan(SADABS; Bruker, 2001)	*h* = −8→10
*T*_min_ = 0.956, *T*_max_ = 0.976	*k* = −8→8
3374 measured reflections	*l* = −16→13

### Refinement

**Table:**

Refinement on *F*^2^	Hydrogen site location: inferred from neighbouring sites
Least-squares matrix: full	H atoms treated by a mixture of independent and constrained refinement
*R*[*F*^2^ > 2σ(*F*^2^)] = 0.052	*w* = 1/[σ^2^(*F*_o_^2^) + (0.0695*P*)^2^] where *P* = (*F*_o_^2^ + 2*F*_c_^2^)/3
*wR*(*F*^2^) = 0.125	(Δ/σ)_max_ < 0.001
*S* = 0.99	Δρ_max_ = 0.25 e Å^−^^3^
2760 reflections	Δρ_min_ = −0.21 e Å^−^^3^
177 parameters	Extinction correction: none
2 restraints	Absolute structure: Flack (1983), 1135 Friedel pairs
Primary atom site location: structure-invariant direct methods	Flack parameter: −0.15 (9)
Secondary atom site location: difference Fourier map	

### Special details

**Table:**

Geometry. All e.s.d.'s (except the e.s.d. in the dihedral angle between two l.s. planes) are estimated using the full covariance matrix. The cell e.s.d.'s are taken into account individually in the estimation of e.s.d.'s in distances, angles and torsion angles; correlations between e.s.d.'s in cell parameters are only used when they are defined by crystal symmetry. An approximate (isotropic) treatment of cell e.s.d.'s is used for estimating e.s.d.'s involving l.s. planes.
Refinement. Refinement of *F*^2^ against ALL reflections. The weighted *R*-factor *wR* and goodness of fit *S* are based on *F*^2^, conventional *R*-factors *R* are based on *F*, with *F* set to zero for negative *F*^2^. The threshold expression of *F*^2^ > σ(*F*^2^) is used only for calculating *R*-factors(gt) *etc*. and is not relevant to the choice of reflections for refinement. *R*-factors based on *F*^2^ are statistically about twice as large as those based on *F*, and *R*- factors based on ALL data will be even larger.

### Fractional atomic coordinates and isotropic or equivalent isotropic displacement parameters (Å^2^)

**Table:**

	*x*	*y*	*z*	*U*_iso_*/*U*_eq_	
Cl1	0.58072 (10)	0.59422 (16)	0.46455 (6)	0.0650 (3)	
O1	0.9356 (2)	1.0348 (4)	0.03436 (15)	0.0508 (6)	
O2	0.7924 (2)	1.2275 (4)	0.12844 (18)	0.0614 (6)	
C1	0.9057 (3)	0.6847 (5)	0.2742 (2)	0.0414 (7)	
H2	0.9376	0.6170	0.2169	0.050*	
C2	0.7841 (3)	0.6008 (5)	0.3216 (2)	0.0439 (7)	
H3	0.7354	0.4763	0.2972	0.053*	
C3	0.7350 (3)	0.7004 (5)	0.4043 (2)	0.0433 (7)	
C4	0.8053 (4)	0.8847 (5)	0.4423 (2)	0.0464 (7)	
H4	0.7703	0.9523	0.4985	0.056*	
C5	0.9290 (3)	0.9666 (5)	0.3950 (2)	0.0432 (7)	
H5	0.9779	1.0902	0.4206	0.052*	
C6	0.9824 (3)	0.8694 (4)	0.3104 (2)	0.0335 (6)	
C7	1.1233 (3)	0.9616 (4)	0.2641 (2)	0.0350 (6)	
H7	1.2084	0.9996	0.3256	0.042*	
C8	1.0761 (3)	1.1622 (4)	0.2018 (2)	0.0333 (6)	
H8	1.0536	1.2665	0.2528	0.040*	
C9	1.2231 (3)	1.2388 (5)	0.1525 (2)	0.0361 (6)	
H9	1.1976	1.3707	0.1159	0.043*	
N10	1.2558 (3)	1.0745 (4)	0.07558 (17)	0.0362 (5)	
H10X	1.157 (2)	1.032 (5)	0.045 (2)	0.039 (8)*	
C11	1.3402 (4)	0.9115 (5)	0.1503 (2)	0.0463 (8)	
H11	1.3969	0.8117	0.1121	0.056*	
C12	1.2061 (4)	0.8080 (4)	0.1992 (2)	0.0413 (7)	
H12A	1.2545	0.6967	0.2454	0.050*	
H12B	1.1233	0.7497	0.1428	0.050*	
C13	1.3871 (3)	1.2488 (5)	0.2315 (3)	0.0478 (8)	
H13A	1.3688	1.2814	0.3027	0.057*	
H13B	1.4592	1.3524	0.2099	0.057*	
C14	1.4630 (3)	1.0344 (6)	0.2289 (2)	0.0532 (8)	
H14A	1.5688	1.0420	0.2057	0.064*	
H14B	1.4797	0.9717	0.2992	0.064*	
C15	0.9197 (3)	1.1414 (4)	0.1143 (2)	0.0377 (6)	
C16	1.3556 (4)	1.1475 (5)	−0.0028 (2)	0.0484 (8)	
H16A	1.3583	1.0429	−0.0557	0.073*	
H16B	1.4664	1.1766	0.0334	0.073*	
H16C	1.3068	1.2702	−0.0369	0.073*	

### Atomic displacement parameters (Å^2^)

**Table:**

	*U*^11^	*U*^22^	*U*^33^	*U*^12^	*U*^13^	*U*^23^
Cl1	0.0584 (5)	0.0864 (7)	0.0526 (4)	−0.0214 (5)	0.0165 (4)	0.0042 (5)
O1	0.0448 (12)	0.0607 (15)	0.0420 (11)	−0.0054 (10)	−0.0048 (9)	−0.0107 (10)
O2	0.0358 (11)	0.0697 (15)	0.0758 (16)	0.0112 (11)	0.0029 (11)	−0.0032 (13)
C1	0.0427 (15)	0.0443 (18)	0.0368 (14)	−0.0029 (13)	0.0060 (12)	−0.0063 (12)
C2	0.0450 (15)	0.0397 (16)	0.0451 (15)	−0.0061 (14)	0.0033 (12)	−0.0024 (14)
C3	0.0341 (14)	0.059 (2)	0.0348 (14)	−0.0067 (13)	0.0003 (12)	0.0086 (14)
C4	0.0455 (17)	0.060 (2)	0.0342 (15)	−0.0006 (15)	0.0076 (12)	−0.0077 (14)
C5	0.0429 (16)	0.0426 (17)	0.0424 (15)	−0.0034 (13)	0.0036 (12)	−0.0102 (14)
C6	0.0327 (14)	0.0343 (15)	0.0311 (13)	0.0039 (11)	−0.0008 (11)	0.0017 (11)
C7	0.0349 (14)	0.0312 (14)	0.0366 (13)	0.0003 (11)	0.0008 (11)	−0.0014 (11)
C8	0.0336 (13)	0.0300 (14)	0.0352 (13)	−0.0006 (10)	0.0035 (11)	−0.0050 (11)
C9	0.0365 (14)	0.0340 (15)	0.0352 (14)	−0.0047 (12)	0.0001 (11)	−0.0025 (12)
N10	0.0327 (11)	0.0390 (13)	0.0359 (11)	−0.0026 (10)	0.0041 (9)	−0.0007 (10)
C11	0.0444 (17)	0.0428 (18)	0.0535 (18)	0.0140 (13)	0.0141 (14)	0.0073 (14)
C12	0.0468 (17)	0.0317 (15)	0.0478 (17)	0.0072 (12)	0.0152 (14)	0.0047 (12)
C13	0.0370 (16)	0.059 (2)	0.0447 (17)	−0.0138 (15)	0.0005 (13)	−0.0033 (15)
C14	0.0316 (14)	0.074 (2)	0.0516 (18)	0.0067 (15)	0.0024 (13)	0.0168 (16)
C15	0.0334 (14)	0.0333 (14)	0.0441 (15)	−0.0019 (11)	0.0012 (12)	0.0058 (12)
C16	0.0463 (16)	0.055 (2)	0.0462 (15)	−0.0037 (14)	0.0140 (13)	0.0037 (14)

### Geometric parameters (Å, °)

**Table:**

Cl1—C3	1.742 (3)	C9—N10	1.506 (4)
O1—C15	1.258 (3)	C9—C13	1.526 (4)
O2—C15	1.229 (3)	C9—H9	0.9800
C1—C2	1.373 (4)	N10—C16	1.482 (3)
C1—C6	1.394 (4)	N10—C11	1.505 (4)
C1—H2	0.9300	N10—H10X	0.881 (17)
C2—C3	1.360 (4)	C11—C14	1.511 (5)
C2—H3	0.9300	C11—C12	1.520 (4)
C3—C4	1.378 (5)	C11—H11	0.9800
C4—C5	1.380 (4)	C12—H12A	0.9700
C4—H4	0.9300	C12—H12B	0.9700
C5—C6	1.388 (4)	C13—C14	1.530 (5)
C5—H5	0.9300	C13—H13A	0.9700
C6—C7	1.517 (4)	C13—H13B	0.9700
C7—C12	1.533 (4)	C14—H14A	0.9700
C7—C8	1.538 (4)	C14—H14B	0.9700
C7—H7	0.9800	C16—H16A	0.9599
C8—C15	1.542 (4)	C16—H16B	0.9599
C8—C9	1.544 (4)	C16—H16C	0.9599
C8—H8	0.9800		
			
C2—C1—C6	121.2 (3)	C11—N10—C9	101.8 (2)
C2—C1—H2	119.4	C16—N10—H10X	112.8 (17)
C6—C1—H2	119.4	C11—N10—H10X	110 (2)
C3—C2—C1	119.7 (3)	C9—N10—H10X	104.4 (19)
C3—C2—H3	120.1	N10—C11—C14	102.7 (3)
C1—C2—H3	120.1	N10—C11—C12	106.7 (2)
C2—C3—C4	121.4 (3)	C14—C11—C12	114.1 (3)
C2—C3—Cl1	119.8 (2)	N10—C11—H11	111.0
C4—C3—Cl1	118.8 (2)	C14—C11—H11	111.0
C3—C4—C5	118.5 (3)	C12—C11—H11	111.0
C3—C4—H4	120.8	C11—C12—C7	111.1 (2)
C5—C4—H4	120.8	C11—C12—H12A	109.4
C4—C5—C6	121.8 (3)	C7—C12—H12A	109.4
C4—C5—H5	119.1	C11—C12—H12B	109.4
C6—C5—H5	119.1	C7—C12—H12B	109.4
C5—C6—C1	117.4 (3)	H12A—C12—H12B	108.0
C5—C6—C7	119.7 (2)	C9—C13—C14	105.1 (3)
C1—C6—C7	122.8 (2)	C9—C13—H13A	110.7
C6—C7—C12	113.6 (2)	C14—C13—H13A	110.7
C6—C7—C8	113.4 (2)	C9—C13—H13B	110.7
C12—C7—C8	111.7 (2)	C14—C13—H13B	110.7
C6—C7—H7	105.8	H13A—C13—H13B	108.8
C12—C7—H7	105.8	C11—C14—C13	105.7 (2)
C8—C7—H7	105.8	C11—C14—H14A	110.6
C7—C8—C15	113.2 (2)	C13—C14—H14A	110.6
C7—C8—C9	109.9 (2)	C11—C14—H14B	110.6
C15—C8—C9	110.2 (2)	C13—C14—H14B	110.6
C7—C8—H8	107.8	H14A—C14—H14B	108.7
C15—C8—H8	107.8	O2—C15—O1	126.1 (3)
C9—C8—H8	107.8	O2—C15—C8	118.2 (3)
N10—C9—C13	102.5 (2)	O1—C15—C8	115.7 (2)
N10—C9—C8	106.5 (2)	N10—C16—H16A	109.5
C13—C9—C8	114.1 (2)	N10—C16—H16B	109.5
N10—C9—H9	111.1	H16A—C16—H16B	109.5
C13—C9—H9	111.1	N10—C16—H16C	109.5
C8—C9—H9	111.1	H16A—C16—H16C	109.5
C16—N10—C11	113.8 (2)	H16B—C16—H16C	109.5
C16—N10—C9	113.5 (2)		
			
C6—C1—C2—C3	1.0 (4)	C13—C9—N10—C16	−77.9 (3)
C1—C2—C3—C4	−0.2 (4)	C8—C9—N10—C16	162.0 (2)
C1—C2—C3—Cl1	−180.0 (2)	C13—C9—N10—C11	44.8 (3)
C2—C3—C4—C5	−0.6 (4)	C8—C9—N10—C11	−75.3 (2)
Cl1—C3—C4—C5	179.2 (2)	C16—N10—C11—C14	77.3 (3)
C3—C4—C5—C6	0.6 (5)	C9—N10—C11—C14	−45.3 (2)
C4—C5—C6—C1	0.2 (4)	C16—N10—C11—C12	−162.4 (2)
C4—C5—C6—C7	−177.7 (3)	C9—N10—C11—C12	75.1 (3)
C2—C1—C6—C5	−1.0 (4)	N10—C11—C12—C7	−62.0 (3)
C2—C1—C6—C7	176.8 (3)	C14—C11—C12—C7	50.7 (3)
C5—C6—C7—C12	160.8 (2)	C6—C7—C12—C11	177.2 (2)
C1—C6—C7—C12	−16.9 (4)	C8—C7—C12—C11	47.4 (3)
C5—C6—C7—C8	−70.3 (3)	N10—C9—C13—C14	−27.1 (3)
C1—C6—C7—C8	112.0 (3)	C8—C9—C13—C14	87.5 (3)
C6—C7—C8—C15	−53.2 (3)	N10—C11—C14—C13	28.0 (3)
C12—C7—C8—C15	76.6 (3)	C12—C11—C14—C13	−87.0 (3)
C6—C7—C8—C9	−177.0 (2)	C9—C13—C14—C11	−0.5 (3)
C12—C7—C8—C9	−47.1 (3)	C7—C8—C15—O2	109.1 (3)
C7—C8—C9—N10	62.1 (2)	C9—C8—C15—O2	−127.3 (3)
C15—C8—C9—N10	−63.3 (3)	C7—C8—C15—O1	−70.6 (3)
C7—C8—C9—C13	−50.1 (3)	C9—C8—C15—O1	53.0 (3)
C15—C8—C9—C13	−175.6 (2)		

### Hydrogen-bond geometry (Å, °)

**Table:**

*D*—H···*A*	*D*—H	H···*A*	*D*···*A*	*D*—H···*A*
N10—H10X···O1	0.881 (17)	1.80 (2)	2.599 (3)	150 (3)
C9—H9···O1^i^	0.98	2.28	3.148 (4)	146
C16—H16A···O2^ii^	0.96	2.48	3.282 (4)	141
C14—H14A···O2^iii^	0.97	2.54	3.439 (4)	154

Symmetry codes:  (i) −*x*+2, *y*+1/2, −*z*; (ii) −*x*+2, *y*−1/2, −*z*; (iii) *x*+1, *y*, *z*.
